# Epidemiological and Clinical Characteristics Associated with Antimicrobial-Resistant Urinary Tract Infections in Outpatient and Inpatient Settings: A Retrospective Study from Northwestern Mexico

**DOI:** 10.3390/pathogens15010092

**Published:** 2026-01-14

**Authors:** Jose Monroy-Higuera, Uriel A. Angulo-Zamudio, Nidia Leon-Sicairos, Hector Flores-Villaseñor, Jorge Velazquez-Roman, Ernesto Ruiz-Trejo, Julio Medina-Serrano, Francisco Castro-Apodaca, Gabriela Tapia-Pastrana, Adrian Canizalez-Roman

**Affiliations:** 1 Programa de Doctorado, Posgrado Integral en Biotecnología, Faculatad de Ciencias Quimico-Biologicas, Universidad Autonoma de Sinaloa, Culiacan 80030, Sinaloa, Mexico; monroyantonio@uas.edu.mx; 2 School of Medicine, Autonomous University of Sinaloa, Culiacan 80019, Sinaloa, Mexico; nidialeon@uas.edu.mx (N.L.-S.); floreshector@uas.edu.mx (H.F.-V.); jorgevelazquezroman@uas.edu.mx (J.V.-R.); 3 Pediatric Hospital of Sinaloa, Culiacan 80200, Sinaloa, Mexico; 4 The Sinaloa State Public Health Laboratory, Secretariat of Health, Culiacan 80058, Sinaloa, Mexico; 5 IMSS Hospital General Regional 1, Culiacan 80230, Sinaloa, Mexico; ernestoruiz93@hotmail.com; 6 Coordinación de Investigación en Salud, Delegacion Instituto Mexicano del Seguro Social, Culiacan 80220, Sinaloa, Mexico; julio.medinase@imss.gob.mx; 7 The Women’s Hospital, Secretariat of Health, Culiacan 80020, Sinaloa, Mexico; francisco.castroapodaca@uas.edu.mx; 8 Laboratorio de Investigación Biomédica, Servicios de Salud del Instituto Mexicano del Seguro Social para el Bienestar (IMSS-BIENESTAR), Hospital Regional de Alta Especialidad de Oaxaca, Oaxaca 71256, Mexico; gtapia@hraeoaxaca.gob.mx

**Keywords:** urinary tract infection, outpatients, inpatients, *E. coli*, antimicrobial resistance, carbapenem resistance

## Abstract

Antimicrobial resistance in urinary tract infections (UTIs) poses a critical public health challenge, yet comparative data between outpatient and inpatient settings remain limited, particularly in Latin America. This study characterized the epidemiology, microbiology, and resistance patterns of UTIs in northwestern Mexico. A retrospective analysis of 1041 patients with UTI (May–November 2024) was conducted. Microorganism identification and antimicrobial susceptibility were determined using the MicroScan WalkAway system in accordance with CLSI guidelines. Results: Outpatients accounted for 80.5% of cases and inpatients for 19.4%, with a 3.1% mortality rate. *Escherichia coli* predominated (62.9%), with a significant association with outpatients (*p* = 0.02), whereas *Enterobacter cloacae*, *Acinetobacter* spp., *Candida tropicalis*, and *C. albicans* were associated with inpatients (*p* < 0.05). Pediatric patients exhibited distinctive microbiological profiles: *Pseudomonas aeruginosa* (9.7% vs. 2.1%, *p* = 0.032), *Enterococcus faecalis* (33.3% vs. 16.2%, *p* = 0.001), and *Staphylococcus epidermidis* (26.6% vs. 6.5%, *p* = 0.027) were significantly more prevalent than in adults. Multidrug resistance (MDR) was detected in 27.1% of isolates, and extensive drug resistance (XDR) in 3.2%. XDR was associated with Gram-positive bacteria (12.2% vs. 1.4%, *p* < 0.001). Carbapenem-resistant *Enterobacteriaceae* (CRE) were identified in 0.9% (7/772) of cases, with 42.9% occurring in outpatients. Hospitalization (OR: 2.01; 95% CI: 1.43–2.83), surgical services (OR: 1.41; 95% CI: 1.02–1.97), and recent surgery (OR: 2.37; 95% CI: 1.04–5.39) were independent predictors of MDR/XDR infections. Conclusions: These findings demonstrate the emergence of CRE within the community and distinctive pediatric resistance patterns, underscoring the need for tailored antimicrobial stewardship strategies in this region.

## 1. Introduction

Urinary tract infections (UTIs) are among the most common diseases worldwide, occurring in both hospitalized and outpatient settings. UTIs are characterized by the growth of microorganisms that cause an inflammatory response in the urinary tract epithelium [1,2]. UTIs account for approximately 150 million cases worldwide each year, and 40% of infections contracted in hospitals are UTIs [3,4]. In Mexico, UTIs are the leading cause of disease in women of reproductive age and the third leading cause of morbidity [5]. UTIs are more prevalent in women than in men. Approximately seven million women suffer from an outpatient UTI each year, and the direct medical costs of UTIs in the United States are estimated to be around $2.19 billion, with an additional $6 billion in costs worldwide [6].

Various microorganisms, including bacteria and fungi, can cause urinary tract infections (UTIs). Bacteria are the most prevalent cause of UTIs, accounting for nearly 95% of cases. The primary etiological agents in UTIs are Gram-negative bacteria, including *Escherichia coli* (*E. coli*), which accounts for approximately 60% of cases, followed by *Klebsiella pneumoniae* (*K. pneumoniae*). Staphylococcus is the most prevalent Gram-positive bacterium, and *Candida* is the most pervasive fungus [7]. These microorganisms are the most common in UTIs in both adults and children.

UTIs in hospitalized or outpatient patients can persist and progress from acute to chronic infections. Several factors may contribute to this phenomenon, but the most critical factor is the microorganism’s antimicrobial resistance [8]. High levels of antibiotic resistance have been reported in *E. coli* isolated from patients with UTIs. For example, resistance to ampicillin ranges from 28% to 40% in countries such as Austria, Greece, Portugal, and the United States. Additionally, high resistance to gentamicin (50%), aminopenicillin+ beta-lactamase inhibitor (50%), ciprofloxacin (45%), and cefuroxime (33%) was observed among *E. coli* isolates from patients with UTIs worldwide between 2003 and 2010 [8]. High levels of antibiotic resistance in UTIs are a public health problem that must be prioritized to treat these infections effectively.

Urinary tract infections (UTIs) are associated with various factors, including poor hygiene and immune system disorders. However, the factors associated with high antibiotic resistance in UTIs are unclear. Identifying the clinical and epidemiological factors associated with antimicrobial resistance in patients with UTIs will help treat infections promptly and accurately, preventing progression to severe or fatal cases. The objective of this study was to: (i) determine the epidemiological and clinical factors of outpatients and inpatients (of all ages) with UTIs; (ii) identify the microorganisms that caused UTIs in outpatients and inpatients and relate them to age groups; (iii) determine the antimicrobial resistance of the microorganisms that caused UTIs; and (iv) associate characteristics of patients with UTIs (epidemiological and clinical) with antimicrobial resistance in northwestern Mexico.

## 2. Materials and Methods

### 2.1. Subjects of Study

This historical, analytical, observational study with a retrospective approach included inpatients and outpatients diagnosed with urinary tract infection (UTI) between May and November 2024 at the Mexican Social Security Institute’s Regional General Hospital No. 1 in northwestern Mexico. UTI was diagnosed based on signs and symptoms, as well as a positive urine culture for a microorganism with 1 × 10^5^ CFU/mL [9,10]. Patients with negative urine cultures and those without a clinical history were excluded. The research was evaluated and approved by the Research Ethics Committee (Local Health Research Committee 2506) of the IMSS Family Medicine Unit No. 46. The registration number is R-2025-2506-045.

### 2.2. Data Collection

Various data were collected from patients’ medical records with UTIs. Epidemiological data included age, sex, demographics, and the hospital department of origin. Clinical data included weight, height, and body mass index (BMI), which was classified according to WHO standards as underweight, normal weight, overweight, or obesity grades I, II, or III [11]. Other clinical data included metabolic diseases (e.g., hypertension, type 2 diabetes, obesity, kidney disease, and dyslipidemia), pregnancy, use of invasive devices, mortality, routine urinalysis, and biochemical analysis.

### 2.3. Microorganisms Isolated from UTI and Antimicrobial Resistance

Urine samples from patients with signs and symptoms of a UTI were processed to isolate microorganisms and determine their antimicrobial resistance. The MicroScan WalkAway 96 automated system (Beckman Coulter, Brea, CA, USA) was used to identify microorganisms and determine antimicrobial resistance according to the manufacturer’s instructions. Gram-positive, Gram-negative, and fungal organisms were isolated from patients, and bacterial and fungal species were identified. Regarding antimicrobial resistance, we tested antibiotics and antifungal agents recommended by the Mexican healthcare system. Antibiotic susceptibility was interpreted according to CLSI guidelines [12], and resistance was classified as follows: in bacteria, resistance to ≥3 distinct antibiotic categories was defined as multidrug resistance (MDR). Bacteria resistant to ≥6 categories of antibiotics were classified as extensively drug-resistant (XDR) [13]. In fungi: MDR were resistant to more than one antifungal agent from two classes, and XDR were resistant to more than one antifungal agent from three classes [14,15].

### 2.4. Statistical Analysis

The Kolmogorov–Smirnov test was used to assess the normality of the data. For quantitative variables, the central tendency (mean) and dispersion (standard deviation and variance) were used. For qualitative variables, percentages and frequencies were used. The chi-square test and binary logistic regression were used in univariate and multivariate analyses to identify associations. A *p*-value of less than 0.05 was considered statistically significant. Based on their distributions, the Kruskal–Wallis test or ANOVA was used for qualitative variables. SPSS version 20 (IBM Corp., Armonk, NY, USA) was used for statistical analysis.

## 3. Results

### 3.1. Epidemiological and Clinical Characteristics of Patients with Urinary Tract Infection

A total of 1041 patients were included in this study, of whom 80.5% (839) were outpatients and 19.4% (202) were inpatients (Table 1). By age group, 4.6% (48/1041) were children (under 18 years of age), 47.7% (497/1041) were adults (18 to 59 years of age), and 45.7% (487/1041) were elderly (60 years of age or older). Among the patients, 76.3% (795/1041) were female, and 23.4% (244/1041) were male. Additionally, 87.9% (879/1041) were from urban areas, and 7.1% (74/1041) were from rural areas. Most urinary tract infections were diagnosed in the family medicine department (36.5%, 380 of 1041), followed by the internal medicine department (29.6%, 309 of 1041). Other departments where UTIs were diagnosed included emergency medicine (12.9%), gynecology, urology, surgery, nephrology, and pediatrics (Table 1). A comparison of outpatients and inpatients revealed that adults, women, and patients from the family medicine and emergency medicine departments were more prevalent among outpatients (*p* ≤ 0.05). Conversely, pediatric patients, older adults, men, and patients from the departments of internal medicine, gynecology, surgery, and traumatology were more prevalent among inpatients (*p* ≤ 0.05).

As shown in Table 2, the clinical characteristics of patients with UTIs are as follows: 3.4% (36/1041) were underweight, 18.7% (195/1041) were normal weight, 36.5% (379/1041) were grade I obese, 8.5% (86/1041) were grade II obese, 7.1% (74/1041) were grade III obese. The most prevalent metabolic disease was hypertension, affecting 43.9% (458/1041) of patients. It was followed by type 2 diabetes (36.5%; 380 patients), obesity (27.9%; 291 patients), kidney disease (20.5%; 214 patients), and dyslipidemia (8.3%; 87 patients). 15.3% (160/1041) of patients were pregnant, and 3.1% (32/1041) died (Table 2). Among patients with urinary tract infections, 10.5% (110/1041) had urinary catheters, and 12.2% (128/1041) had undergone surgery within the past month. Thirty-four percent (316/1041) of patients had leukocyte esterase present in their urine analysis, 28.6% (298/1041) had nitrites, 15.8% (165/1041) had protein, 3.9% (41/1041) had ketones, 18.1% (188/1041) had erythrocytes, 23.9% (249/1041) had 10–85 leukocytes, 14.6% (153/1041) had moderate to abundant epithelial cells, 32% (334/1041) had moderate to abundant bacteria, and 13.9% (145/1041) had hematuria. The primary biochemical characteristics of patients with urinary tract infections are shown in Table 2 and are as follows: creatinine, 1.42 mg/dL; blood urea nitrogen, 15.5 mg/dL; leukocytes, 6.84%; erythrocytes, 3.34%; hemoglobin, 9.49 g/dL; hematocrit, 28.3%; lymphocytes, 18.4%; and neutrophils, 47.4%.

Some clinical characteristics were associated with outpatient status. For instance, grade I obesity and metabolic diseases were related to outpatients in the BMI category. Overall, the presence of nitrites in a urine analysis was associated with outpatients (*p* ≤ 0.05; see Table 2). Urinary catheter use, prior surgeries, and mortality were associated with inpatients (*p* ≤ 0.05; see Table 2). Most general urine analysis parameters were more frequently observed in inpatients (except for nitrites and moderate-to-abundant epithelial cells). Low biochemical characteristics of erythrocytes, hemoglobin, hematocrit, lymphocytes, and neutrophils were also associated with hospitalized patients (*p* ≤ 0.05; see Table 2).

### 3.2. Distribution of the Primary Microorganisms That Cause Urinary Tract Infections in Hospitalized and Outpatient Patients

Table 3 shows the microorganisms isolated from outpatients and inpatients with urinary tract infections. Of these, 80.5% (839 out of 1041) were Gram-negative bacteria, 16.5% (172 out of 1041) were Gram-positive bacteria, and 2.9% (30 out of 1041) were fungi. Among the Gram-negative bacteria, *E. coli* was the most prevalent (62.9%, or 526 out of 839), followed by *K. pneumoniae* (17.1%, or 144 out of 839), *Proteus mirabilis* (*P. mirabilis*) (9.7%, or 82 out of 839), *Enterobacter cloacae* (*E. cloacae)* (2.5%, or 21 out of 839), *Pseudomonas aeruginosa* (*P. aeruginosa*) (2.1%, or 10 out of 839), and *Citrobacter* and *Acinetobacter* (1.1%, or 10 out of 839) The most prevalent Gram-positive bacteria were *Streptococcus haemolyticus* (*S. haemolyticus*) (22.9%, 39/172), *Enterococcus faecalis* (*E. faecalis)* (21.5%, 37/172), *Streptococcus agalactiae* (*S. agalactiae*) (11.6%, 20/172), *Staphylococcus aureus* (*S. aureus*) (9.3%, 16/172), and *Staphylococcus epidermidis* (*S. epidermidis*) (8.1%, 14/172) (Table 3). Among fungi, *Candida tropicalis* (*C. tropicalis*) was the most prevalent (36.6%, 11/30), followed by *Candida albicans* (*C. albicans*) (30%, 9/30), *Candida glabrata* (*C. glabrata*) (23.3%, 7/30), and *Candida krusei* (*C. krusei*) (6.6%, 2/30). Additionally, 11.8% (123/1041) of cases were polymicrobial (Table 3). The presence of *E. coli* was associated with outpatients (*p* ≤ 0.05). Gram-negative bacteria (*E. cloacae* and *Acinetobacter* spp.), fungi (*C. tropicalis* and *C. albicans*), and hospitalization were generally associated (*p* ≤ 0.05; Table 3). The distribution of Gram-positive bacteria and polymicrobial infections was similar between outpatient and inpatient settings.

Additionally, the distribution of microorganisms by age group was analyzed (see Table 4). Most microorganisms were evenly distributed among the three age groups: pediatric, adult, and elderly. However, *P. aeruginosa* was more prevalent in the pediatric group than in the adult group (9.7% vs. 2.1%, *p* = 0.032) and the elderly group (9.7% vs. 2.1%, *p* = 0.023). Gram-positive bacteria were generally more prevalent in the pediatric age group than in the geriatric age group (31.2% vs. 6.9%, *p* = 0.001). *E. faecalis* (33.3% vs. 16.2%, *p* = 0.001) and *S. epidermidis* (26.6% vs. 6.5%, *p* = 0.027) were more prevalent in the pediatric group than in the adult group.

### 3.3. Antimicrobial Resistance of Microorganisms Isolated from Patients with Urinary Tract Infections

Table 5 presents antimicrobial resistance data for Gram-negative and Gram-positive bacteria. Overall, 70.2% (710/1011) of the bacteria were resistant to at least one antimicrobial. Of those, 27.1% (274/1011) were multidrug-resistant (MDR), and 3.2% (33/1011) were extensively drug-resistant (XDR). Twenty-nine-point seven percent (301/1011) of the bacteria were sensitive to antibiotics, 66.8% (709/1011) were resistant to one to six antimicrobials, and 33.2% (302/1011) were resistant to seven to 13 antimicrobials (Table 5). A comparison of Gram-negative and Gram-positive bacteria revealed that the XDR category was associated with Gram-positive bacteria (12.2% vs. 1.4%, *p* < 0.001). The second and third antimicrobials were associated with Gram-negative bacteria (20.9% vs. 6.3%, *p* < 0.001, and 19.3% vs. 4%, *p* < 0.001, respectively). The fourth, fifth, sixth, and ninth antimicrobials were also associated with Gram-negative bacteria (9.8% vs. 0.5%, *p* < 0.001; 8.1% vs. 0.1%, *p* < 0.001; 6.9% vs. 0.1%, *p* < 0.001; and 2.3% vs. 0.1%, *p* = 0.003). Only Gram-negative bacteria were resistant to 10–13 antibiotics (Table 5).

In addition, antimicrobial resistance was analyzed in outpatient and hospital settings (see Supplementary Table S1). Hospitalized patients were more likely to be resistant to at least one antimicrobial agent (80.6% vs. 70.1%, *p* = 0.004), multidrug resistant (MDR) (39.2% vs. 24.4%, *p* < 0.001), and extensively drug resistant (XDR) (7.3% vs. 2.3%, *p* = 0.002). Outpatients were more likely to be sensitive to antimicrobials (31.9% vs. 19.3%, *p* < 0.001) and to one antimicrobial agent (22.6% vs. 15.3%, *p* = 0.033). However, hospitalized patients were more likely to have three (23.8% vs. 15.2%, *p* = 0.007), six (3.4% vs. 1%, *p* = 0.032), or seven (3.9% vs. 0.7%, *p* = 0.002) antimicrobial resistances.

### 3.4. Carbapenem-Resistant Enterobacteriaceae

Carbapenem resistance was identified in 7 of 772 (0.9%) *Enterobacteriaceae* isolates, including four *E. coli* (57.1%), two *K. pneumoniae* (28.6%), and one *P. mirabilis* (14.3%). The median age of patients with Carbapenem-resistant Enterobacteriaceae (CRE) was 61 years (range 39–80), with male predominance (71.4%). In the clinical setting, four cases (57.1%) occurred in hospitalized patients, whereas three cases (42.9%) were identified among outpatients attending primary care (Family Medicine) or Internal Medicine consultations. Among comorbidities, diabetes mellitus was present in two patients (28.6%), hypertension in three (42.9%), and chronic kidney disease in one (14.3%). All CRE isolates were classified as MDR, with a high fluoroquinolone co-resistance rate of 83.3% (5/6 tested). No mortality was observed among CRE cases. Detailed characteristics of these patients are presented in Supplementary Table S2.

### 3.5. Epidemiological and Clinical Factors in Patients with Urinary Tract Infections Associated with Antimicrobial Resistance

We analyzed the epidemiological and clinical factors of patients with urinary tract infections according to four categories of antimicrobial resistance in microorganisms: susceptible (n = 301), three or fewer categories (n = 403), MDR (n = 274), and XDR (n = 33) (see Supplementary Table S3). Epidemiological and clinical factors associated with three or fewer categories included advanced age and the hospital’s surgical department. Older adults, internal medicine, surgery, type 2 diabetes, urinary catheter use, mortality, and the presence of leukocyte esterase, nitrites, protein, erythrocytes, and leukocytes (10–85 U) in the general urine analysis were associated with MDR infection. The following were associated with XDR infection: being an adult or male; receiving services from the nephrology, pediatrics, or traumatology departments; having a low body weight; having kidney disease; having had previous surgery; being pregnant; and having protein, ketones, erythrocytes, and hematuria in a general urine analysis. Next, binary logistic regression was used to assess the association between epidemiological and clinical factors and antimicrobial resistance in patients with UTIs, as shown in Table 6. Hospitalization (OR: 2.01; 95% CI: 1.43–2.83; *p* < 0.001) and the surgery department (OR: 1.41; 95% CI: 1.02–1.97; *p* = 0.049) were associated with multidrug resistance. Hospitalization (OR: 2.87; 95% CI: 1.40–5.86; *p* = 0.003) and previous surgery (OR: 2.37; 95% CI: 1.04–5.39; *p* = 0.038) were associated with resistance to multiple antimicrobial drugs.

## 4. Discussion

A urinary tract infection (UTI) is one of the most prevalent infectious diseases worldwide. Several microorganisms can cause UTIs, and most of them are highly resistant to antibiotics. Depending on the severity of the infection, patients may be hospitalized or treated as outpatients, and specific characteristics may be associated with these states or antibiotic resistance. In this study, we demonstrated that the following factors were associated with outpatients and inpatients: epidemiological characteristics (age, sex, and hospital department) and clinical characteristics (BMI, metabolic disease, use of invasive devices, and general urine analysis parameters). UTI mortality in this study was 3.1%. Gram-negative bacteria, such as *E. coli* and *K. pneumoniae*, were the most frequently isolated microorganisms. Furthermore, *E. coli* was associated with outpatients, while *E. Cloacae, Acinetobacter* spp., *C. tropicalis*, and *C. albicans* were associated with inpatients. Additionally, UTIs caused by *P. aeruginosa, E. faecalis*, and *S. epidermidis* were more prevalent among pediatric patients. Gram-positive bacteria were more resistant to antibiotics than Gram-negative bacteria. Microorganisms isolated from hospitalized patients were more resistant than those from outpatients. Finally, patient characteristics, such as hospital department (e.g., surgery), prior surgeries, and hospitalizations, were associated with multidrug-resistant (MDR) and/or extensively drug-resistant (XDR) infections.

This study found that some epidemiological characteristics, such as age, sex, and hospital department, were more prevalent among outpatients and inpatients, consistent with other studies [16,17,18]. Regarding clinical characteristics, grade II obesity was associated with urinary tract infections (UTIs) in outpatients. However, the relationship between obesity and UTIs is controversial. Some studies indicate that obesity increases the likelihood of developing UTIs because increased central fat in men may contribute to benign prostatic hyperplasia [19,20,21]; In contrast, Nassaji et al. (2015) found no association between UTIs and BMI in adults, suggesting that obesity is not a risk factor for UTIs [22]. The relationship between obesity and outpatients does not align with findings from other studies. For example, García-Bustos et al. (2021) found that obesity was a predictor of increased hospital stay due to UTI [23]. However, obesity is a risk factor not only for hospitalization due to UTIs but also for other diseases, such as liver disease and COVID-19 [23,24]. The relationship between obesity and outpatients with UTIs in this study may be due to Mexico’s high obesity rate, as it is among the world leaders in obesity [25,26]. Other clinical characteristics observed among patients hospitalized for UTIs included the use of invasive devices and alterations in most general urine test parameters. This is because UTIs that require hospitalization tend to be more aggressive than those that do not.

It is important to note that the association between nitrite positivity and outpatient status should be interpreted in the context of the differential distribution of uropathogens between settings. Nitrite production requires bacterial nitrate reductase activity, an enzyme present in *Enterobacteriaceae* such as *E. coli* and *K. pneumoniae*, but absent in *Enterococcus* spp., *Staphylococcus* spp., *Pseudomonas aeruginosa, Acinetobacter* spp., and *Candida species*. The significantly higher prevalence of *E. coli* in outpatients (63.1% vs. 43.0%, *p* = 0.02) and the association of non-nitrite-producing organisms (*E. cloacae, Acinetobacter* spp., *C. tropicalis, C. albicans*) with hospitalized patients explains this finding rather than representing a diagnostic limitation. Furthermore, the high prevalence of urinary catheterization in inpatients (38.1% vs. 3.9%) reduces bladder dwell time, decreasing the opportunity for nitrate-to-nitrite conversion. Regarding epithelial cells, no significant difference was observed between groups (*p* = 0.842), and all diagnoses were confirmed by quantitative urine culture (≥10^5^ CFU/mL) as the reference standard.

The 3.1% mortality rate from urinary tract infections in this study is consistent with rates found in other studies. For instance, Cornejo-Dávila (2015) reported a 2.9% mortality rate from urinary tract infections in Mexico [27]. Gharbi et al. (2019) reported that the mortality rate from urinary tract infections ranged from 1.6% to 5.4% among patients aged 65 years or older in England [28].

However, our mortality rate is lower than that reported in other studies. For instance, Kitagawa et al. (2019) found a mortality rate of 14.5% in patients with a urinary tract infection and bacteremia caused by *P. aeruginosa* in Japan [29]. Álvarez-Artero et al. (2021) found a 16.5% mortality rate in Spain for urinary tract infections caused by *Enterococcus* spp. [30]. In terms of microbiology, *E. coli* and *K. pneumoniae* were the most prevalent bacteria in isolates from outpatients and inpatients with UTIs, consistent with other studies [30,31,32]. When analyzing the distribution of UTI-causing microorganisms separately between outpatients and inpatients, E. coli was more commonly identified among outpatients. This result coincides with other studies [26,33,34]. In contrast to other studies, Gram-negative bacteria such as *E. cloacae* and *Acinetobacter* spp., as well as the fungi *C. tropicalis* and *C. albicans*, were associated with hospitalized UTIs. These bacteria and fungi were previously associated with UTIs in hospitalized patients [35,36,37]. In children, *P. aeruginosa, E. faecalis*, and *S. epidermidis* were associated with infection [36,38]. Unlike other studies, we demonstrated not only the prevalence of the main microorganisms causing UTIs in hospitalized and outpatient patients, as well as in children, adults, and older adults, but also a possible association between the presence of these microorganisms and these study groups. However, in some cases, the microorganisms we identified in association with subjects’ conditions or ages differ from those reported as the most prevalent in other studies. The prevalence of microorganisms that cause UTIs (excluding *E. coli* and *K. pneumoniae*) may vary by country, subjects’ microbiota, socioeconomic status, or prior antibiotic use [6,39,40].

Our findings revealed a distinctive microbiological profile in pediatric UTI patients that warrants special attention. *P. aeruginosa* was 4.6-fold more prevalent in children compared to adults and older adults (9.7% vs. 2.1%, *p* = 0.032), while *E. faecalis* was twice as common (33.3% vs. 16.2%, *p* = 0.001) and *S. epidermidis* was four times more frequent (26.6% vs. 6.5%, *p* = 0.027). These findings are consistent with international reports. Bitsori et al. demonstrated that *P. aeruginosa* UTI in children is strongly associated with prior antibiotic use (OR 21.6, 95% CI 4.65–100), previous UTI episodes, and urinary tract malformations [41]. Similarly, the European Society for Pediatric Infectious Diseases (ESPID) guidelines emphasize that *P. aeruginosa* and *Enterococcus* spp. are more common in children with recurrent UTIs, urological abnormalities, or prior antibiotic exposure [42].

The clinical relevance of these findings lies in the implications for empirical therapy selection. Standard empirical regimens for pediatric UTI, typically third-generation cephalosporins, do not provide adequate coverage against *Enterococcus* spp. or *P. aeruginosa* [43,44]. Our data suggest that clinicians in northwestern Mexico should consider broadening empirical coverage for pediatric patients with recurrent UTIs or known urological abnormalities.

Regarding antibiotic resistance, high levels have been reported in the microorganisms that cause urinary tract infections. Fenta et al. (2020) demonstrated that 66% of urinary tract infections isolated from children in Ethiopia were multidrug-resistant, primarily caused by Gram-negative bacteria [45]. In Nepal, Shrestha et al. (2021) found that 89% of urinary tract infections were multidrug-resistant, with 29.2% resistant to four classes, 21.5% to five classes, and 7.6% to six or more classes [46]. Huang et al. (2022) found that bacteria isolated from urinary tract infections were multidrug-resistant in 0–91.6% and extremely multidrug-resistant in 0–30.4% of cases in China between 2009 and 2020 [36]. The antibiotic resistance rates in these studies are higher than those found in northwestern Mexico. In our study, we identified that 27.1% of the bacteria causing UTIs were multidrug-resistant (MDR) and 3.2% were extensively drug-resistant (XDR).

Additionally, we found that the XDR phenotype was associated with Gram-positive bacteria and that MDR and XDR infections were associated with patients hospitalized for UTIs. However, we found that other countries had similar or lower levels of resistance. For instance, Weber et al. (2025) found that 22% of urinary tract infection (UTI) isolates were multidrug resistant (MDR) in Germany between 2013 and 2022 [47]. In the United States, Ku et al. (2024) reported that 13% of UTI-causing strains were MDR, with 3% resistant to four classes of antibiotics, 1% to five classes, and less than 1% to six classes [48]. Although other countries have higher levels, antibiotic resistance in strains isolated from urinary tract infections was high. The high prevalence of antibiotic resistance could be related to the indiscriminate use of antibiotics in Mexico across clinical practice, food production, and veterinary medicine [48,49,50].

One of the most concerning findings of this study was the identification of CRE in 0.9% of urinary isolates. Although this prevalence appears relatively low compared to that in endemic regions, the clinical and epidemiological significance of this finding cannot be overstated. The World Health Organization classifies CRE as a critical priority pathogen due to minimal therapeutic options and high mortality rates associated with these infections [51].

Particularly alarming was the finding that 42.9% of CRE cases (3/7) occurred in outpatients attending primary care or Internal Medicine consultations, rather than in hospitalized patients with traditional healthcare-associated risk factors. This pattern suggests community dissemination of carbapenem-resistant organisms in northwestern Mexico, a phenomenon increasingly reported in Latin America but rarely documented in Mexican ambulatory settings [52,53]. The presence of CRE in community-dwelling patients without recent hospitalization represents a paradigm shift in the epidemiology of these critical pathogens. It poses significant challenges for empirical antibiotic selection in the outpatient management of UTI. The detection of CRE in outpatients also highlights the importance of obtaining urine cultures before initiating empirical therapy, particularly in patients with recurrent UTIs or prior antibiotic exposure.

Finally, some patient characteristics, such as surgery, prior surgery, and hospitalization, were associated with multidrug-resistant and/or extremely resistant infections. Other studies have also associated patient characteristics with antibiotic resistance in patients with urinary tract infections. For example, Chen et al. (2013) found that urinary catheter use and kidney stones among men were associated with cefazolin resistance in urinary tract infections [54]. Faine et al. (2015) identified three clinical factors associated with multidrug-resistant urinary tract infections in the United States: male sex, chronic hemodialysis, and nursing home residence [55]. He et al. (2024) found that intensive care unit (ICU) stays, including prolonged ICU stays, were associated with ESBL-associated urinary tract infections in children [55]. Identifying characteristics associated with resistance-related urinary tract infections in Mexico will help identify cases at risk of infection by highly antibiotic-resistant microorganisms. With this knowledge, strategies can be developed to promptly treat cases of urinary tract infections complicated by highly antibiotic-resistant organisms.

The distinction between uncomplicated and complicated (or localized and systemic) UTIs has essential implications for the selection of empiric therapy and its duration. Recent guidelines from the EAU (2025) and IDSA (2025) have refined UTI classification to focus on clinical presentation rather than underlying host factors alone [56,57,58]. Although our study did not prospectively apply these classification systems, the significant differences observed between outpatients and inpatients—including higher mortality (14.8% vs. 0.2%), greater catheter use (38.1% vs. 3.9%), and more frequent isolation of healthcare-associated pathogens in inpatients—suggest that hospitalized patients likely had more severe or complicated infections. The higher prevalence of non-*E. coli* organisms, including *Candida species* and *Acinetobacter* spp., among inpatients further supports this interpretation, as pathogen diversity is a recognized feature of complicated UTIs.

This study has several limitations. First, because of its retrospective design, UTIs could not be formally classified according to current frameworks (e.g., uncomplicated/complicated or localized/systemic). The classification of UTI severity requires prospective documentation of specific symptoms, vital signs, and imaging findings that were inconsistently available in medical records. However, several clinical variables in our dataset serve as proxies for infection severity, including hospitalization status, ICU admission, catheter use, mortality, and biochemical parameters. Second, UTIs were not differentiated by anatomical site (lower vs. upper tract) due to the lack of systematic imaging. Third, blood cultures were not routinely obtained, precluding identification of bacteremic cases. Finally, the retrospective design limited access to longitudinal outcome data and to prior antimicrobial treatment data, both of which are recognized risk factors for antimicrobial-resistant UTIs. Future prospective studies in this region should incorporate standardized UTI classification systems, such as the EAU 2025 localized/systemic framework, to better characterize infection severity and guide targeted antimicrobial stewardship interventions [7,59,60]. In addition, prospective studies should systematically capture antibiotic exposure history to better characterize resistance risk factors in this population.

This is the first comprehensive study to report the distribution of primary microorganisms causing UTIs among hospitalized patients, outpatients, and children. Additionally, antibiotic resistance and the characteristics of patients with UTIs associated with antibiotic resistance were analyzed.

## 5. Conclusions

This study provides evidence that specific epidemiological, clinical, and biochemical characteristics of patients are associated with UTIs in hospitalized and outpatient settings. Mortality in northwestern Mexico was 3.1%. The main microorganisms causing UTIs were *E. coli* and *K. pneumoniae*. Some bacteria or fungi were associated with hospitalized patients, outpatients, and children. Additionally, high levels of antibiotic resistance were observed among Gram-positive bacteria and other microorganisms infecting hospitalized patients. The identification of carbapenem-resistant Enterobacteriaceae in both inpatient and outpatient settings signals the community emergence of last-resort antibiotic resistance in northwestern Mexico, warranting urgent implementation of enhanced surveillance and antimicrobial stewardship programs. Finally, patient characteristics, including hospital department (e.g., surgery), prior surgeries, and length of hospitalization, were associated with urinary tract infections caused by multidrug-resistant (MDR) and/or extensively drug-resistant (XDR) microorganisms. These results will help medical staff in our region diagnose urinary tract infections faster in hospitalized patients, outpatients, and children. Additionally, we will be able to provide optimal treatment and prevent mortality from urinary tract infections in patients of all ages. Respecting the identified risk factors will help develop strategies to prevent urinary tract infections caused by multidrug-resistant or extremely drug-resistant microorganisms.

## Figures and Tables

**Table 1:** Epidemiological Characteristics of Inpatient and Outpatient Patients with Urinary Tract Infections.

Characteristics	Total	Outpatient n (%)	Inpatient n (%)	*p*-Value *
	n = 1041	n = 839 (80.5)	n = 202 (19.4)	
Age groups				
Pediatrics (<18 years)	48 (4.6)	33 (0.4)	15 (7.4) *	0.03
Adults (18–59 years)	497 (47.7)	428 (51.0) *	69 (34.1)	<0.001
Older adult (≥60 years)	487 (46.7)	369 (43.9)	118 (58.4) *	<0.001
Sex, n (%)				
Female	795 (76.3)	653 (77.8) *	142 (70.2)	0.02
Male	244 (23.4)	184 (21.9)	60 (29.7) *	0.02
Not determined	23 (0.1)	23 (2.7)	0 (0.0)	
Demography				
Urban	915 (87.9)	734 (87.4)	181 (89.6)	0.104
Rural	74 (7.1)	58 (6.9)	16 (7.9)	0.647
Not determined	52 (4.9)	47 (5.6)	5 (2.4)	-
Hospital service				
Family medicine	380 (36.5)	376 (44.8) *	4 (1.9)	<0.001
Internal medicine	309 (29.6)	221 (26.3)	88 (43.5) *	<0.001
Emergency Department/ICU	135 (12.9)	119 (14.1) *	16 (7.9)	0.019
Gynecology and Obstetrics	63 (6.0)	35 (4.1)	28 (13.8) *	<0.001
Urology	36 (3.4)	22 (2.6)	14 (6.9)	0.005
Not determined	35 (3.3)	35 (4.1)	0 (0.0)	-
Surgery	23 (2.2)	3 (0.3)	20 (9.9) *	<0.001
Nephrology	22 (2.1)	16 (1.9)	6 (2.9)	0.41
Pediatrics	20 (1.9)	6 (0.7)	14 (6.9) *	<0.001
Traumatology and Orthopedics	18 (1.7)	6 (0.7)	12 (5.9) *	<0.001

Statistical significance was obtained using Fisher’s exact test. *p* value *: ≤0.05 statistically significant.

**Table 2:** Clinical Characteristics of Outpatient and Inpatient Patients with Urinary Tract Infections.

Characteristics	Total	Outpatient n (%)	Inpatient n (%)	*p*-Value *
	n = 1041	n = 839 (80.5)	n = 202 (19.4)	
BMI category				
Low weight	36 (3.4)	26 (3.0)	10 (4.9)	0.196
Normal weight (18.5–24.9)	195 (18.7)	158 (18.8)	37 (18.3)	0.866
Overweight (25–29.9)	380 (36.5)	308 (36.7)	72 (35.6)	0.777
Obesity grade I (30–34.9)	199 (19.1)	174 (20.7) *	25 (12.3)	0.007
Obesity grade II (35–39.9)	89 (8.5)	72 (8.5)	17 (8.4)	0.94
Obesity grade III (≥40)	74 (7.1)	62 (7.3)	12 (5.9)	0.472
Not determined	68 (6.5)	39 (4.6)	29 (14.3)	-
Metabolic disease				
High blood pressure	458 (43.9)	358 (42.6)	100 (49.5)	0.079
Type 2 diabetes	380 (36.5)	295 (35.1)	85 (42.1)	0.067
Obesity	291 (27.9)	252 (30.0) *	39 (19.3)	0.002
Kidney disease	214 (20.5)	164 (19.5)	50 (24.7)	0.167
Dyslipidemia	87 (8.3)	75 (8.9)	12 (5.9)	0.1 00
Pregnancy	160 (15.3)	132 (15.7)	28 (19.2)	0.508
Invasive devices				
Bladder catheter	110 (10.5)	33 (3.9)	77 (38.1) *	<0.001
Previous Surgery (last month)	128 (12.2)	76 (9.0)	52 (25.7) *	<0.001
Mortality	32 (3.1)	2 (0.2)	30 (14.8) *	<0.001
General Urine Test				
Leukocyte esterase	316 (30.4)	228 (27.2)	88 (43.6) *	<0.001
Nitrites	298 (28.6)	260 (31.0) *	38 (18.8)	<0.001
Proteins	165 (15.8)	97 (11.5)	68 (33.7) *	<0.001
Ketones	41 (3.9)	13 (1.2)	28 (13.9) *	<0.001
Erythrocytes	188 (18.1)	104 (12.4)	84 (41.6) *	<0.001
Leukocytes 10 to 85 U	249 (23.9)	170 (13.0)	79 (41.6) *	<0.001
Moderate to abundant epithelial cells	153 (14.6)	125 (12.0)	28 (13.9)	0.842
Moderate to abundant bacterial presence	334 (32.0)	243 (17.3)	91 (45.0) *	<0.001
Hematuria	145 (13.9)	74 (8.8)	71 (35.1) *	<0.001
Biochemical traits—Media (SD)				
Creatinine (mg/dL)	1.42 ± 8.6	1.33 ± 8.34	1.79 ± 9.6	0.758
Blood uric nitrogen (mg/dL)	15.5 ± 20.3	14.6 ± 16.8	21.04 ± 3.4	0.248
Leukocytes (%)	6.84 ± 4.9	6.65 ± 4.1	7.62 ± 7.7	0.739
Erythrocytes (%)	3.34 ± 2.8	3.41 ± 2.5	3.07 ± 4.0 *	<0.001
Hemoglobin (g/dL)	9.49 ± 6.5	9.88 ± 9.72	7.89 ± 5.1 *	<0.001
Hematocrit (%)	28.3 ± 16.1	29.31 ± 16.1	24.2 ± 15.7 *	<0.001
Lymphocytes (%)	18.4 ± 34.6	20.6 ± 37.8	9.28 ± 12.4 *	<0.001
Neutrophils (%)	47.4 ± 28.7	47.1 ± 26.9	48.8 ± 35.5 *	0.001

Abbreviations: g: grams, dL: deciliter, mg: milligrams, ng, nanograms, L: liters, %: percentage, U: units. Normal value: creatinine: 0.5–12 mg/dL, Blood uric nitrogen 6–20 mg/dL, erythrocytes 4.2–5.4%, hemoglobin: 12–16 gr/dL, lymphocytes: 24–38%, neutrophils 55–70%. Statistical significance was obtained using Fisher’s exact test. * *p* value: ≤0.05 statistically significant.

**Table 3:** Distribution of the Primary Microorganisms Causing Urinary Tract Infections in Outpatient and Inpatient Patients.

Microorganisms	Total	Outpatient n (%)	Inpatient n (%)	*p*-Value *
	n = 1041	n = 839 (80.5)	n = 202 (19.4)	
Gram-negative bacteria	840 (80.5)	694 (70.0)	145 (71.7) *	0.001
*Escherichia coli*	526 (62.9)	438 (63.1) *	87 (43.0)	0.02
*Klebsiella pneumoniae*	144 (17.1)	118 (17.0)	26 (12.8)	0.659
*Proteus mirabilis*	82 (9.7)	64 (9.2)	18 (8.9)	0.255
*Enterobacter cloacae*	21 (2.5)	10 (1.4)	8 (3.9) *	0.029
*Pseudomonas aeruginosa*	18 (2.1)	17 (2.4)	4 (1.9)	0.967
*Citrobacter* spp.	10 (1.1)	8 (1.1)	2 (0.9)	0.962
*Acinetobacter* spp.	10 (1.1)	5 (0.7)	5 (2.4) *	0.014
Gram-positive bacteria	172 (16.5)	141 (16.8)	31 (15.3)	0.616
*Staphylococcus haemolyticus*	39 (22.9)	33 (23.4)	6 (19.5)	0.518
*Enterococcus faecalis*	37 (21.5)	30 (21.7)	7 (22.5)	0.858
*Streptococcus agalactiae*	20 (11.6)	19 (13.4)	1 (3.2)	0.1 00
*Staphylococcus aureus*	16 (9.3)	13 (9.2)	3 (9.6)	0.947
*Staphylococcus epidermidis*	14 (8.1)	10 (7.0)	4 (12.9)	0.383
Fungus	30 (2.9)	4 (0.5)	26 (12.8) *	<0.001
*Candida tropicalis*	11 (36.6)	1 (25.0)	11 (42.3) *	<0.001
*Candida albicans*	9 (30.0)	1 (25.0)	8 (30.7) *	<0.001
*Candida glabrata*	7 (23.3)	0 (0.0)	7 (26.9)	-
*Candida krusei*	2 (6.6)	2 (50.0)	0 (0.0)	-
Polymicrobial infections	123 (11.8)	98 (11.6)	25 (12.3)	0.738

Other *Gram-negative bacteria* found in patients: *Proteus mirabilis*, *Serratia marcescens*, *Serratia fonticola*, *Klebsiella oxytoca*, *Pseudomonas luteola*, *Enterobacter aerogenes*, *Streptococcus salivarius*, *Proteus penneri*, *Morganella morganii*, *Providencia stuartii*, *Enterobacter aerogenes*, *Pseudomonas putida*, *Providencia rettgeri*, and *Pantoea* spp. Other Gram-positive bacteria found in patients: *Staphylococcus saprophyticus*, *Streptococcus mitis*, *Staphylococcus hominis*, *Staphylococcus sciuri*, *Enterococcus faecium*, *Kocuria kristinae*, *Kocuria varians*, *Enterococcus casseliflavus*, *Streptococcus sanguinis*, *Streptococcus dysgalactiae*, *α-hemolytic Streptococcus*, *Streptococcus uberis*, *Streptococcus thoraltensis*, *Streptococcus suis*, and *Streptococcus sanguinis*. Other fungi found in patients: *Candida* spp. Statistical significance was obtained using Fisher’s exact test. *p* value *: ≤0.05 statistically significant.

**Table 4:** Distribution of Microorganisms Causing Urinary Tract Infections by Age Group.

Microorganisms	Total	Age Group				
		Pediatric < 18 Years n (%)	Adult 18–59 Years n (%)	Older Adult ≥ 60 Years n (%)	*p*-Value *	
	n = 1041	n = 48 (4.6)	n = 497 (47.7)	n = 487 (46.7)	Pediatric vs. Adult	Pediatric vs. Old Adult
Gram-negative bacteria	839 (80.5)	41 (85.4)	369 (74.2)	429 (88.9)	0.113	0.614
*Escherichia coli*	526 (62.9)	26 (63.4)	228 (61.7)	272 (63.4)	0.837	0.879
*Klebsiella pneumoniae*	144 (17.1)	7 (17.0)	73 (19.7)	64 (14.9)	0.188	0.202
*Proteus mirabilis*	82 (9.7)	4 (9.7)	33 (8.9)	45 (10.4)	0.745	0.662
*Pseudomonas aeruginosa*	21 (2.5)	4 (9.7) *#	8 (2.1)	9 (2.1)	0.032	0.023
*Enterobacter cloacae*	18 (2.1)	0 (0.0)	10 (2.7)	8 (1.8)	-	-
*Citrobacter* spp.	10 (1.1)	0 (0.0)	1 (0.2)	9 (2.0)	-	-
*Acinetobacter* spp.	10 (1.1)	0 (0.0)	3 (0.8)	7 (1.6)	-	-
Gram-positive bacteria	172 (16.5)	15 (31.2) #	123 (24.7)	34 (6.9)	0.384	0.001
*Enterococcus faecalis*	39 (3.7)	5 (33.3) *	20 (16.2)	14 (41.1)	0.001	0.753
*Staphylococcus haemolyticus*	37 (3.5)	4 (26.6)	27 (21.9)	6 (17.6)	0.744	0.47
*Streptococcus agalactiae*	20 (1.9)	1 (6.6)	15 (12.1)	4 (11.7)	0.1 00	0.141
*Staphylococcus aureus*	16 (1.5)	1 (6.6)	13 (10.5)	2 (5.8)	0.1 00	0.1 00
*Staphylococcus epidermidis*	14 (1.3)	4 (26.6) *	8 (6.5)	2 (5.8)	0.027	0.062
Fungus	30 (2.9)	1 (2.0)	5 (1.0)	24 (5.1)	0.426	0.716
*Candida tropicalis*	11 (36.6)	0 (0.0)	2 (40.0)	9 (37.5)	-	-
*Candida albicans*	9 (30.0)	1 (100)	1 (20.0)	7 (29.1)	0.333	0.08
*Candida glabrata*	7 (23.3)	0 (0.0)	1 (20.0)	6 (25.0)	-	-
*Candida krusei*	2 (6.6)	0 (0.0)	1 (20.0)	1 (4.1)	-	-
Polymicrobial infections	123 (11.8)	2 (4.1)	59 (11.8)	62 (12.7)	0.147	0.101

Statistical significance was obtained using Fisher’s exact test. *: ≤0.05 statistically significant pediatric vs. adult; #: ≤0.05 statistically significant pediatric vs. older adult.

**Table 5:** Antimicrobial Resistance of Gram-Negative and Gram-Positive Bacteria Isolated from Patients with Urinary Tract Infections.

Category	Total Bacteria	Gram-Negative n (%)	Gram-Positive n (%)	*p* Value
	n = 1011 (%)	n = 839 (76.6)	n = 172 (23.4)	
At least one antimicrobial	710 (70.2)	592 (70.5)	118 (68.6)	0.782
MDR	274 (27.1)	218 (25.9)	56 (32.5)	0.089
XDR	33 (3.2)	12 (1.4)	21 (12.2) *	<0.001
Resistance by number of antimicrobials				
0	301 (29.7)	247 (29.4)	54 (31.9)	0.647
1	216 (21.3)	186 (22.1)	30 (17.4)	0.185
2	187 (18.4)	176 (20.9) *	11 (6.3)	<0.001
3	169 (16.7)	162 (19.3) *	7 (4.0)	<0.001
4	70 (6.9)	50 (5.9)	20 (11.6)	0.466
5	22 (2.1)	5 (0.5)	17 (9.8) *	<0.001
6	15 (1.4)	1 (0.1)	14 (8.1) *	<0.001
7	13 (1.2)	1 (0.1)	12 (6.9) *	<0.001
8	3 (0.2)	0 (0.0)	3 (1.4)	-
9	5 (0.4)	1 (0.1)	4 (2.3) *	0.003
10	1 (0.1)	1 (0.1)	0 (0.0)	-
11	4 (0.3)	4 (0.4)	0 (0.0)	-
12	4 (0.3)	4 (0.4)	0 (0.0)	-
13	1 (0.1)	1 (0.1)	0 (0.0)	-

Of the total patients infected by bacteria (n = 1042), one lacked an antibiogram, which was a Gram-negative bacterium. MDR: multidrug resistant, XDR: extremely resistant. The classification of resistance of bacteria was carried out based on the article by [14], while the classification of fungi was by [15,16]). Statistical significance was obtained using Fisher’s exact test. *p* value *: ≤0.05 statistically significant.

**Table 6:** Characteristics of Patients with Urinary Tract Infections Associated with Antibiotic Resistance.

Characteristics of UTI Patients	Type of Resistance	OR	CI 95%	*p* Value
Hospital services				
Surgery	MDR	1.41	1.02–1.97	0.049
Inpatient	MDR	2.01	1.43–2.83	<0.001
	XDR	2.87	1.40–5.86	0.003
Invasive devices				
Previous Surgery (last month)	XDR	2.37	1.04–5.39	0.038

Abbreviation: OR: odds ratio; CI: confidence interval. Binary logistic regression under a multivariate model was used to get associations. Only the statistically significant characteristics of the patients with UTI are shown.

## Data Availability

The original contributions presented in this study are included in the article/Supplementary Material. Further inquiries can be directed to the corresponding authors.

## Supplementary Materials

The following supporting information can be downloaded at: https://www.mdpi.com/article/10.3390/pathogens15010092/s1. Table S1: Antimicrobial Resistance in Outpatients and Inpatients; Table S2: Clinical and microbiological characteristics of patients with carbapenem-resistant Enterobacteriaceae urinary tract infections (n = 7); Table S3: Epidemiological and Clinical Factors Related to Antibiotic Resistance in Patients with Urinary Tract Infection.
