# Supplementary material for: Epidemiological and Clinical Characteristics Associated with Antimicrobial-Resistant Urinary Tract Infections in Outpatient and Inpatient Settings: A Retrospective Study from Northwestern Mexico
Source: Pathogens. 2026 Jan 14;15(1):92. doi: 10.3390/pathogens15010092 (PMC12845216; doi:10.3390/pathogens15010092)
Supplement: Supplementary file 1 [file pathogens-15-00092-s001.zip › pathogens-4064268-supplementary.pdf]

Supplementary Table S1. Antimicrobial Resistance in Outpatients and Inpatients.

Category	Total microorganisms n= 1011 (%)	Outpatient n (%) n=835 (80.5)	Inpatient n (%) n=176 (19.4)	*p* value
At least one antimicrobial	710 (70.2)	586 (70.1)	142 (80.6)*	0.004
MDR	274 (27.1)	205 (24.4)	69 (39.2)*	<0.001
XDR	33 (3.2)	20 (2.3)	13 (7.3)*	0.002
Resistance by number of antimicrobials				
0	301 (29.7)	267 (31.9)*	34 (19.3)	<0.001
1	216 (21.3)	189 (22.6)*	27 (15.3)	0.033
2	187 (18.4)	154 (18.4)	33 (18.7)	0.915
3	169 (16.7)	127 (15.2)	42 (23.8)*	0.007
4	70 (6.9)	52 (6.20)	18 (10.2)	0.07
5	22 (2.1)	18 (2.1)	4 (2.2)	0.1 00
6	15 (1.4)	9 (1.0)	6 (3.4)*	0.032
7	13 (1.2)	6 (0.7)	7 (3.9)*	0.002
8	3 (0.2)	3 (0.3)	0 (0.0)	-
9	5 (0.4)	5 (0.5)	0 (0.0)	-
10	1 (0.1)	1 (0.1)	0 (0.0)	-
11	4 (0.3)	2 (0.2)	2 (1.1)	0.142
12	4 (0.3)	2 (0.2)	2 (1.1)	0.142
13	1 (0.1)	0 (0.0)	1 (0.5)	-

Of the total samples (n=1041), 30 samples lacked an antibiogram, of which four were from outpatients and 26 were inpatient, MDR: multidrug resistant, XDR: extremely resistant. The classification of resistance of bacteria was carried out based on the article by Magiorakos AP et al. (2012), while the classification of fungi was by Arendrup & Patterson (2017) and Jacobs et al. (2022). Statistical significance was obtained using Fisher's exact test. P value *: ≤0.05 statistically significant.

Supplementary Table S2. Clinical and microbiological characteristics of patients with carbapenem-resistant *Enterobacteriaceae* urinary tract infections (n=7).

Case	Microorganism	Age	Sex	Setting	DM	HTN	CKD	Service	MEM	ETP	CIP	AMK	Resistance	Outcome
**1**	*E. coli*	39	F	Inp	No	No	No	Surgery	R	R	R	ND	MDR	Alive
**2**	*K. pneumoniae*	80	M	Inp	Yes	Yes	No	Internal Med	R	S	R	S	MDR	Alive
**3**	*E. coli*	77	M	Out	No	Yes	No	Family Med	S	R	S	S	MDR	Alive
**4**	*P. mirabilis*	61	M	Out	No	No	No	Family Med	S	R	ND	R	MDR	Alive
**5**	*E. coli*	53	M	Inp	No	No	No	Internal Med	R	S	R	S	MDR	Alive
**6**	*K. pneumoniae*	39	F	Out	No	No	No	Internal Med	S	R	R	S	MDR	Alive
**7**	*E. coli*	67	M	Inp	Yes	Yes	Yes	Internal Med	R	R	R	S	MDR	Alive

Abbreviations: DM, diabetes mellitus; HTN, hypertension; CKD, chronic kidney disease; MEM, meropenem; ETP, ertapenem; CIP, ciprofloxacin; AMK, amikacin; MDR, multidrug-resistant; Inp, inpatient; Out, outpatient; R, resistant; S, susceptible; ND, not determined; Family Med, Family Medicine; Internal Med, Internal Medicine.

Supplementary Table S3. Epidemiological and Clinical Factors Related to Antibiotic Resistance in Patients with Urinary Tract Infection.

Characteristics of UTI patients	Susceptible n=301 (%)	<3 categories n=403 n (%)	MDR n=274 n (%)	XDR n=33 n (%)	p value
**Epidemiological**					
**Age groups**					
Pediatrics (<18 years)	8 (2.6)	22 (5.4)	13 (4.7)	3 (9.0)	0.239
Adults (18-59 years)	176 (58.4)	178 (44.1)	124 (45.2)	20 (60.6)*	<0.001
Older adult (≥60 years)	116 (35.8)	201 (49.5)*	136 (49.6)*	10 (30.3)	<0.001
**Sex**					
Female	258 (85.7)*	309 (76.6)	185 (67.5)	22 (66.6)	<0.001
Male	41 (13.6)	94 (23.3)	89 (32.4)*	11 (33.3)*	<0.001
**Hospital service**					
Family medicine	130 (43.1)*	153 (37.9)	90 (32.8)	5 (15.5)	<0.001
Internal medicine	86 (28.7)	107 (26.5)	92 (33.5)*	7 (21.2)	0.016
Emergency Department/ICU	33 (10.9)	60 (14.8)	38 (13.8)	2 (6.0)	0.275
Gynecology and Obstetrics	24 (7.9)	24 (5.9)	10 (3.6)	4 (12.2)	0.144
Urology	8 (2.6)	15 (3.7)	11 (4.0)	2 (6.0)	0.496
Surgery	3 (0.9)	8 (1.9)*	5 (1.8)*	0 (0.0)	<0.001
Nephrology	2 (0.6)	7 (1.7)	8 (2.9)	5 (15.1)*	<0.001
Pediatrics	6 (1.9)	6 (1.4)	11 (4.0)	3 (9.0)*	<0.001
Traumatology and Orthopedics	0 (0.0)	5 (1.2)	3 (1.0)	2 (6.0)*	0.03
**Clinical**					
**BMI category**					
Low weight	3 (0.9)	17 (4.2)	10 (3.6)	5 (15.1)*	0.001
Normal weight (18.5-24.9)	49 (16.2)	82 (20.3)	53 (19.3)	6 (18.1)	0.729
Overweight (25-29.9)	104 (34.5)	142 (35.2)	108 (39.4)	18 (54.5)	0.105
Obesity grade I (30-34.9)	66 (21.9)	78 (19.3)	49 (17.8)	1 (3.0)	0.113
Obesity grade II (35-39.9)	34 (11.2)	33 (8.1)	19 (6.9)	1 (3.0)	0.257
Obesity grade III (≥40)	21 (6.9)	33 (8.1)	16 (5.8)	0 (0.0)	0.225
**Metabolic disease**					
High blood pressure	132 (43.8)	167 (41.4)	133 (48.5)	11 (33.3)	0.255
Type 2 diabetes	96 (31.8)	145 (35.9)	111 (40.5)*	11 (33.3)	0.039
Obesity	94 (31.2)	101 (25.0)	80 (29.1)	9 (27.2)	0.432
Kidney disease	43 (14.2)	86 (21.3)	72 (26.2)	10 (30.3)*	0.002
Dyslipidemia	30 (9.9)	26 (6.4)	25 (9.1)	3 (9.0)	0.514
**Invasive devices**					
Bladder catheter	14 (4.6)	32 (7.9)	46 (16.7)*	2 (6.0)	<0.001
Previous Surgery (last month)	26 (8.6)	36 (8.9)	47 (17.1)	10 (30.3)*	<0.001
**Pregnancy**	61 (20.2)	57 (14.4)	30 (10.9)	10 (30.3)*	0.002
**Mortality**	2 (0.6)	8 (1.9)	12 (4.3)*	1 (3.0)	<0.001
**General Urine Test**					
Leukocyte esterase	78 (25.9)	115 (28.5)	97 (35.4)*	9 (27.3)	0.011
Nitrites	46 (15.3)	65 (16.1)	50 (18.2)*	4 (12.1)	0.001
Proteins	30 (10.0)	58 (14.4)	57 (20.8)*	6 (18.2)*	<0.001
Ketones	8 (2.7)	9 (2.2)	16 (5.8)	3 (9.1)*	<0.001
Erythrocytes	35 (11.6)	64 (15.9)	62 (23.0)*	7 (21.2)*	<0.001
Leukocytes 10 to 85%	60 (19.9)	87 (21.6)	78 (28.5)*	7 (21.2)	<0.001
Moderate to abundant epithelial cells	48 (15.9)	57 (14.1)	36 (13.1)	5 (15.2)	0.175
Moderate to abundant bacterial presence	86 (28.6)	126 (31.3)	97 (35.4)	11 (33.3)	0.256
Hematuria	25 (8.3)	50 (12.4)	46 (16.8)	8 (24.2)*	<0.001
**Biochemical traits**					
Creatinine (mg/dL)	0.73 ± 1.0	1.0 ± 1.3	2.34 ± 14.4*	1.0 ± 1.5	0.035
Blood uric nitrogen (mg/dL)	11.6 ± 13.3	17.2 ± 22.4	17.1 ± 20.4	18.0 ± 26.9	0.065
Leukocytes (%)	6.8 ± 4.2	6.6 ± 4.7	6.6 ± 5.8	7.3 ± 6.1	0.184
Erythrocytes (%)	3.2 ± 1.8	3.4 ± 3.1	3.2 ± 3.0	3.7 ± 5.9	0.8
Hemoglobin (gr/dl)	10.5 ± 8.6	9.6 ± 5.2	8.9 ± 5.3	8.3 ± 5.5	0.07
Hematocrit (%)	29.0 ± 15.9	29.1 ± 16.0	26.8 ± 16.5	25.3 ± 16.6	0.092
Lymphocytes (%)	18.5 ± 14.3	18.33 ± 15.8	20.6 ± 62.7	12.7 ± 12.3	0.095
Neutrophils (%)	48.3 ± 27.5	47.1 ± 27.5	46.1 ± 30.2	49.2 ± 34.2	0.35

Abbreviations: g: grams, dL: deciliter, mg: milligrams, ng: nanograms, fL: femtoliter, L: liters, U: units, %: percentage. MDR: multidrug resistant, XDR: extremely resistant. The classification of resistance of bacteria was carried out based on the article by Magiorakos AP et al. (2012), while the classification of fungi was by Arendrup & Patterson (2017) and Jacobs et al. (2022). Statistical significance was obtained using Kruskal Wallis, and Mann Whitney tests to get statistical significance. *: ≤0.05.

1. Magiorakos, A.P.; Srinivasan, A.; Carey, R.; Carmeli, Y.; Falagas, M.; Giske, C.; Harbarth, S.; Hindler, J.F.; Kahlmeter, G.; Olsson-Liljequist, B.; et al. Multidrug-resistant, extensively drug-resistant and pandrug-resistant bacteria: An international expert proposal for interim standard definitions for acquired resistance. *Clin. Microbiol. Infect.* **2012**, *18*, 268–281.
2. Arendrup, M.C.; Patterson, T.F. Multidrug-Resistant Candida: Epidemiology, Molecular Mechanisms, and Treatment. *J. Infect. Dis.* **2017**, *216*, S445‑S451. https://doi.org/10.1093/infdis/jix131.
3. Jacobs, S.E.; Jacobs, J.L.; Dennis, E.K.; Taimur, S.; Rana, M.; Patel, D.; Gitman, M.; Patel, G.; Schaefer, S.; Iyer, K.; et al. C*andida auris* Pan-Drug-Resistant to Four Classes of Antifungal Agents. *Antimicrob. Agents Chemother.* **2022**, *66*, e0005322. https://doi.org/10.1128/aac.00053-22.
